# In Silico Structural and Functional Characterization of HtrA Proteins of *Leptospira* spp.: Possible Implications in Pathogenesis

**DOI:** 10.3390/tropicalmed5040179

**Published:** 2020-11-28

**Authors:** Brenda Bevilaqua Daroz, Luis Guilherme Virgílio Fernandes, Aline Florencio Teixeira, Ana Lucia Tabet Oller Nascimento

**Affiliations:** 1 Laboratório Especial de Desenvolvimento de Vacinas, Instituto Butantan, São Paulo 05503-000, Brazil; brenda.daroz@butantan.gov.br (B.B.D.); luis.fernandes@butantan.gov.br (L.G.V.F.); aline.teixeira@butantan.gov.br (A.F.T.); 2 Programa de Pós-Graduação Interunidades em Biotecnologia, Instituto de Ciências Biomédicas, São Paulo 05508-900, Brazil

**Keywords:** *Leptospira*, leptospirosis, HtrA protein, pathogenesis

## Abstract

Leptospirosis is a zoonosis caused by the pathogenic bacteria of the genus *Leptospira*. The identification of conserved outer membrane proteins among pathogenic strains is a major research target in elucidating mechanisms of pathogenicity. Surface-exposed proteins are most probably the ones involved in the interaction of leptospires with the environment. Some spirochetes use outer membrane proteases as a way to penetrate host tissues. HtrA is a family of proteins found in various cell types, from prokaryotes to primates. They are a set of proteases usually composed of a serine protease and PDZ domains, and they are generally transported to the periplasm. Here, we identified four genes—annotated as HtrA, LIC11111, LIC20143, LIC20144 and LIC11037—and another one annotated as a serine protease, LIC11112. It is believed that the last forms a functional heterodimer with LIC11111, since they are organized in one operon. Our analyses showed that these proteins are highly conserved among pathogenic strains. LIC11112, LIC20143, and LIC11037 have the serine protease domain with the conserved catalytic triad His-Asp-Ser. This is the first bioinformatics analysis of HtrA proteins from *Leptospira* that suggests their proteolytic activity potential. Experimental studies are warranted to elucidate this possibility.

## 1. Introduction

Leptospirosis is an important zoonosis caused by the pathogenic bacteria of the genus *Leptospira*, affecting humans and animals worldwide. The disease is responsible for causing more than 1 million infections with 60,000 deaths per year [1]. Human infection occurs mainly through direct contact with the urine or other biological fluids of infected animals or via indirect contact with contaminated soil or water [2,3]. After contact with damaged skin or mucosa, leptospires can rapidly penetrate and overcome host biological barriers, reaching target organs within 1 hour of infection [4], showing high invasive potential. 

The disease displays several clinical manifestations, ranging from asymptomatic, mild, and benign, to more severe forms. The initial phase is characterized by fever, chills, headache and muscle pain, and it can progress to a more severe condition, known as Weil’s syndrome. This condition is characterized by hemorrhage, renal failure, and jaundice. Often, the initial symptoms can be confused with other diseases, such as influenza and dengue, making it difficult to diagnose and possibly leading to the patient’s death [5].

The genus *Leptospira* includes helical-shaped bacteria, and phylogenetic analysis has revealed that the genus can be divided into three clades associated with the level of pathogenicity of the species: saprophytic (7), intermediate (5), and pathogenic (10) [6]. Besides genetic classification, leptospires can also be serologically divided, regarding serogroup and serovar status, associated with the antigenic heterogeneity of exposed lipopolysaccharides (LPS) [3]. The most frequent serovar detected in humans in Brazil is *L. interrogans* serovar Copenhageni [7]. In 2004, Nascimento and colleagues [8] sequenced the genome of this strain and observed the presence of two chromosomes. Chromosome I has 4,277,185 bp, while chromosome II contains 350,181 bp. The number of genes found in this genome reflects the ability of *Leptospira* to respond to diverse stimuli.

The identification of genes encoding conserved outer membrane or secreted proteins among pathogenic strains is a major research goal to elucidate pathogenicity mechanisms. Some spirochetes utilize proteases as a mechanism of dissemination in host tissues. *Leptospira* cells are able to subvert host cell functions, especially the fibrinolytic system, to enhance their invasiveness. By capturing circulating plasminogen (PLG) and inducing the endothelial secretion of its activator, leptospires can generate plasmin (PLA) and thereby become proteolytically active bacteria. The in vitro degradation of extracellular matrix (ECM) components, immune modulators and coagulation factors has been shown by PLA-coated leptospires [9,10]. 

Besides the exogenously acquired degradation potential, some endogenous proteases have also been demonstrated in pathogenic *Leptospira*, such as collagenases [11,12], thermolysins [13,14,15], metalloproteases [16], and oligopeptidases [17]. However, by data-mining the available genome sequences, it was observed that endogenous proteolytic potential remains unexplored. Proteins of the high temperature requirement A (HtrA) family can be found in various cell types, from prokaryotes to primates. They are a set of proteases generally transported to the periplasm, where they form proteolytic active oligomers with an important function in protein quality control [18,19]. They usually possess a trypsin-like serine protease catalytic domain, near the N-terminal and one or two PDZ domains (named after the first three proteins in which the domain was observed; post-synaptic density protein 95, *Drosophila* disc large tumor suppressor, and zonula occludens 1) at the C-terminal, a sequence tag responsible for protein-protein interactions [20,21]. Bacterial HtrA proteases are responsible for removing improperly folded proteins. The abnormal folded proteins are normally found in harsh conditions such as extreme pH, elevated temperature and increased oxidative or osmotic stress [22]. For a long time, it was believed that HtrA proteases were uniquely functional within the bacterial periplasm [23]. However, aside from their housekeeping roles, the recently identified secreted fractions of HtrAs can directly contribute to bacterial pathogenesis, an activity described for *Campylobacter jejuni* [24] and *Helicobacter pylori* [25,26]. These HtrA proteins can be actively secreted into the extracellular environment, where they are able to cleave host cell factors. *Borrelia burgdorferi* is a spirochetal bacterium that is the etiological agent of Lyme disease. *B. burgdorferi* possesses an HtrA (HtrABb) that has already been studied by several laboratories, and it is clear that this protease has multiple and diverse proteolytic and chaperone functions [27,28]. These roles include the degradation of ECM components such as fibronectin, e-cadherin, and proteoglycans [27] and several endogenous proteins [29]. Accordingly, it is reasonable to predict that these proteins could play a similar role in *Leptospira* spp.

Five genes annotated as probable HtrA protein-coding genes are found in the genome of *L. interrogans* serovar Copenhageni: LIC11111, LIC11112, and LIC11037 (chromosome I); and LIC20143 and LIC20144 (chromosome II). They have been recently re-annotated as LIC_RS05730, LIC_RS05735, LIC_RS05355, LIC_RS18720, and LIC_RS18725, respectively. The proteins encoded by the genes LIC11111 and LIC20144 are annotated as HtrA2 and HtrA1-like protein, respectively, and they only possess the PDZ domain; on the other hand, the proteins encoded by the genes LIC11112, LIC11037, and LIC20143 are serine protease, HtrA- periplasmic trypsin-like serine protease and HtrA1-serine protease, respectively. Unlike LIC11112, which does not have the PDZ sequence, LIC11037 and LIC20143 code for PDZ and trypsin-like serine protease domains. Although these five proteins described so far are annotated as HtrA proteins, we believe that only LIC20143 and LIC11037 code for true HtrA proteins because they bear both the serine protease and PDZ domain. This study aimed to determine the in silico characteristics of these five HtrA proteins in different *Leptospira* species and to discuss their possible role in virulence.

## 2. Methods

### 2.1. HtrAs Sequence Analysis Among *Leptospira* spp. and Functional Characterization

The LIC11111, LIC11112, LIC11037, LIC20143, and LIC20144 coding sequences (CDSs) were selected from the *L. interrogans* serovar Copenhageni genome [8,30]. Amino-acid sequences of potential HtrAs orthologs from 20 species of *Leptospira* were retrieved from the NCBI database through the Basic Local Alignment Search Tool (BLAST) [31], specifically the protein-protein BLAST (BLASTp) analysis against the non-redundant protein sequence database. For comparison purposes, the following species were considered: *L. interrogans* serovar Copenhageni L1-130 (taxid:44275), *L. kirschneri* (taxid:29507), *L. noguchii* (taxid:28182)*, L. alstonii* (taxid:28452)*, L. weilii* (taxid:28184)*, L. alexanderi* (taxid:100053)*, L. borgpetersenii* (taxid:174)*, L. santarosai* (taxid:28183)*, L. kmetyi* (taxid:408139)*, L. fainei* (taxid:48782)*, L. broomii* (taxid:301541)*, L. wolffii* (taxid:409998), *L. licerasiae* (taxid:447106), *L. inadai* (taxid:29506), *L. wolbachii* (taxid:29511), *L. yanagawae* (taxid:293069), *L. biflexa* (taxid:172), *L. vanthielii* (taxid:293085), *L. terpstrae* (taxid:293075), and *L. meyeri* (taxid:29508). In addition, conservation analysis was performed for all five genes studied. Table S1 contains all the Sequence IDs used in this study.

These protein sequences were analyzed using the TMHMM server v.2.0 [32] and the SMART program [33] for prediction of transmembrane-helix and conserved domain, respectively. The CELLO program [34] was used to predict protein localization and LipoP to search for the signal peptide cleavage site. The DOOR 2.0 program was used to search for genes in operons [35].

### 2.2. Protein Alignment

The phylogenetic relationships were constructed with sequences obtained in this study (Table S1), using the maximum-likelihood method and JTT model [36], using MegaX software [37,38]. Node support for the resulting phylogenetic tree was evaluated by 1000 bootstrap replicates to determine the robustness of the finding [39]. Aiming to search for the conserved amino acids in the serine protease domain present in the LIC20143, LIC11112, and LIC11037 an alignment was performed by using the PROMALS3D alignment program [40]. 

### 2.3. Three-Dimensional (3D) Structure and the Search for Conserved Domains

To investigate the position of the catalytic triad and PDZ regions in the structure of the proteins coded by LIC11111, LIC11112, LIC20143, LIC20144, and LIC11037, the 3D structure of these proteins was modeled by submitting the linear sequences to the I-TASSER [41] web server. I-TASSER web server generated a total of five models of each target protein. The models with the best confidence score and Z-score were chosen and then visualized using the PyMOL molecular graphics system for all five sequences. Zooming the probable catalytic triads, also using PyMOL software, shows the predicted positions of the three amino acids.

## 3. Results

### 3.1. Conservation Analysis in Leptospiral Strains

**Figure 1:**
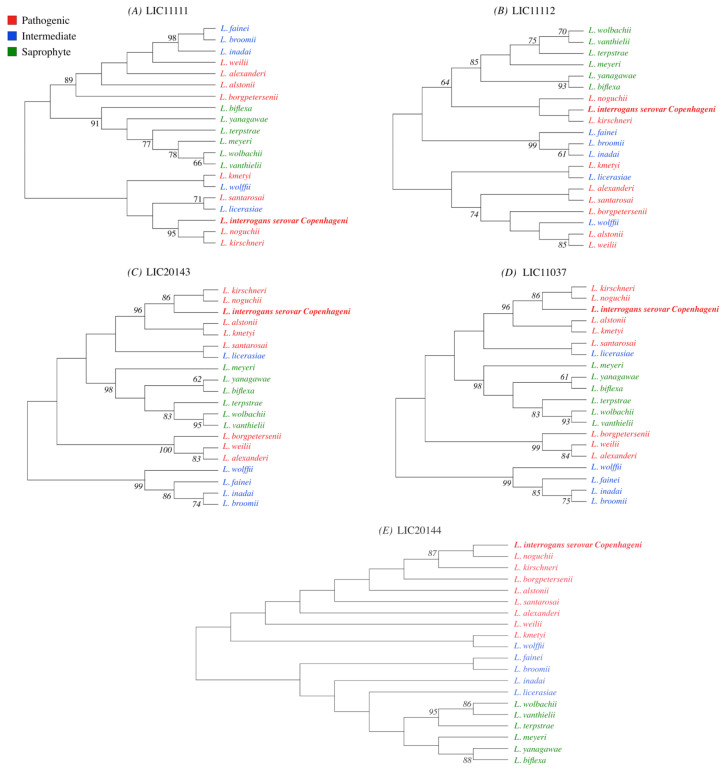
Evolutionary analysis by maximum likelihood method among *Leptospira* strains for (**A**) LIC11111, (**B**) LIC11112, (**C**) LIC20143, (**D**) LIC11037, and (**E**) LIC20144. Amino acids sequences from proteins encoded by the five genes were analyzed in silico by BLASTp with sequences available in the GenBank database and were used to perform evolutionary history by using the maximum likelihood method on MegaX. The phylograms generated show a high degree of identity between pathogenic species, whereas the saprophytic species presented a lower conservation of the target sequences. The percentage of trees in which the associated taxa clustered together in the bootstrap test (1000 replicates) is shown next to the branches, only values higher than 60 were considered.

The CDS of each HtrA protein was selected from the genome of *L. interrogans* serovar Copenhageni based on in silico analysis (Table S1). The evolutionary history for LIC11111, LIC11112, LIC20143, LIC20144, and LIC11037 proteins was inferred by using the maximum likelihood method. The trees with the highest log likelihood are shown (Figure 1). The percentage of replicate trees in which the associated taxa clustered together in the bootstrap test (1000 replicates) are shown next to the branches, only percentages higher than 60 were considered. Initial trees for the heuristic search were obtained automatically by applying Neighbor-Join and BioNJ algorithms to a matrix of pairwise distances estimated using the JTT model, and then selecting the topology with superior log likelihood value. This analysis was performed with amino acid sequences from 20 pathogenic, intermediate and saprophyte species of *Leptospira* for each gene. Evolutionary analyses were conducted in MEGA X [37,38]. The saprophyte group (shown in green) for all five proteins share a common evolutionary ancestor with high bootstrap percentage. Interestingly, some intermediate (shown in blue) share a history with pathogenic (shown in red) strains, for example *L. santarosai* and *L. licerasiae* for LIC11111 share a direct common ancestor. *L. interrogans*, *L. noguchii*, and *L. kirschneri* also share a common ancestor for all five genes analyzed.

### 3.2. Distribution of HtrAs of Pathogenic, Intermediate, and Saprophytic *Leptospira* Strains

**Figure 2:**
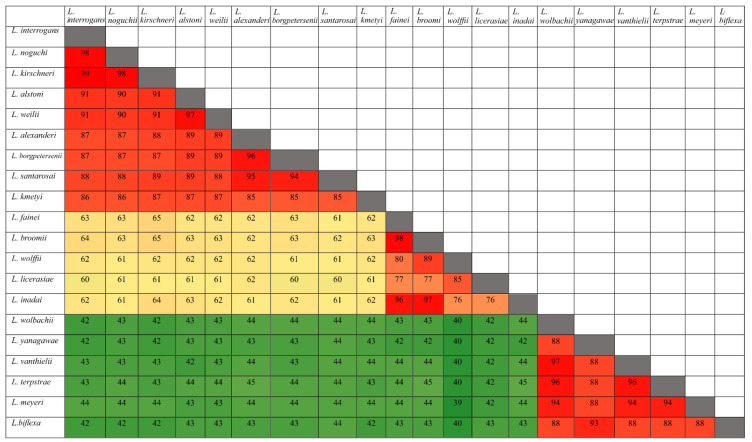
Amino acid sequence identities of LIC11112 from 20 different *Leptospira* strains. The image was generated comparing the sequence of the protein encoded by LIC11112 from *L. interrogans* serovar Copenhageni with other 20 species (pathogenic, intermediate, and saprophytic). Similarity is shown in red above 80%, in yellow between 50% and 80% and in green below 50%.

**Figure 3:**
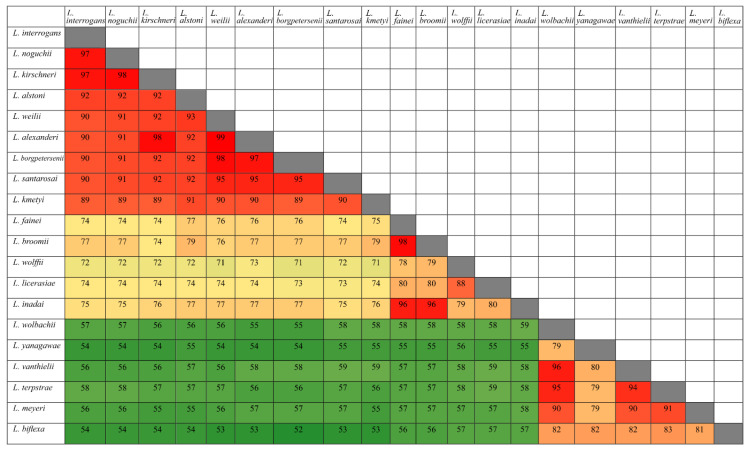
Amino acid sequence identities of LIC20143 from 20 different *Leptospira* strains. The image was generated comparing the sequence of the protein encoded by LIC20143 from *L. interrogans* serovar Copenhageni with other 20 species (pathogenic, intermediate, and saprophytic). Similarity is shown in red above 80%, in yellow between 50% and 80%, and in green below 50%.

To determine how conserved amino acid sequences of HtrAs are among leptospiral groups (pathogenic, intermediate, and saprophytic), we carried out an extensive study using BLAST analysis. The five identified HtrAs sequences (LIC11111, LIC11112, LIC11037, LIC20143, and LIC20144) were submitted to GenBank and compared with the sequence database of 20 selected species of *Leptospira* [42]. We compared amino acid sequences, crossing each species with each other. When comparison was within the same leptospiral group, similarity was high, as shown in Figure 2 and Figure 3 for LIC11112 and LIC20143, respectively. However, when the pathogenic species were compared with the intermediate and saprophytic species, similarities decreased. This indicated that these proteins were highly conserved in their own group. The same analysis was performed with LIC11111, LIC11037, and LIC20144 proteins, and the results are shown in Figures S1–S3, respectively.

### 3.3. Features of the HtrA Proteins

To better investigate the five genes encoding HtrA proteins, a compilation of characteristics was generated depicting: strains, amino acid number and sequence of SPI cleavage sites, transmembrane regions, amino acids corresponding to serine protease, PDZ domain location within the amino acid number, size in amino acids and cellular localization (Table 1). Four species of different groups were selected to do this analysis: *L. interrogans* and *L. borgpetersenii* (pathogenic), *L. licerasiae* (intermediate), and *L. biflexa* (saprophytic). As we can see in Table 1, both LIC11111 and LIC11112 have cleavage sites in all four species studied, while LIC11037 only in *L. biflexa.* For LIC20143 and LIC20144 the cleavage sites in all species except *L. borgptersenii* and *L. biflexa,* respectively. The sequence in which the cleavage occurs is also specified in Table 1. All the genes except LIC11111 and LIC20144 possess a serine protease domain, being that for LIC11112 these domains are also absent in the intermediate and saprophyte species. The in silico analysis for cellular localization of the proteins predicted that all of them, except LIC11037 and LIC20144 in *L. biflexa,* that showed localization at the outer membrane. All proteins possess a PDZ domain with the exception of LIC11111 in *L. biflexa*, LIC11112 in *L. interrogans* and *L. borgpetersenii*, and LIC20144 in *L. biflexa.*

### 3.4. 3D Structure Model and Domain Architecture

**Figure 4:**
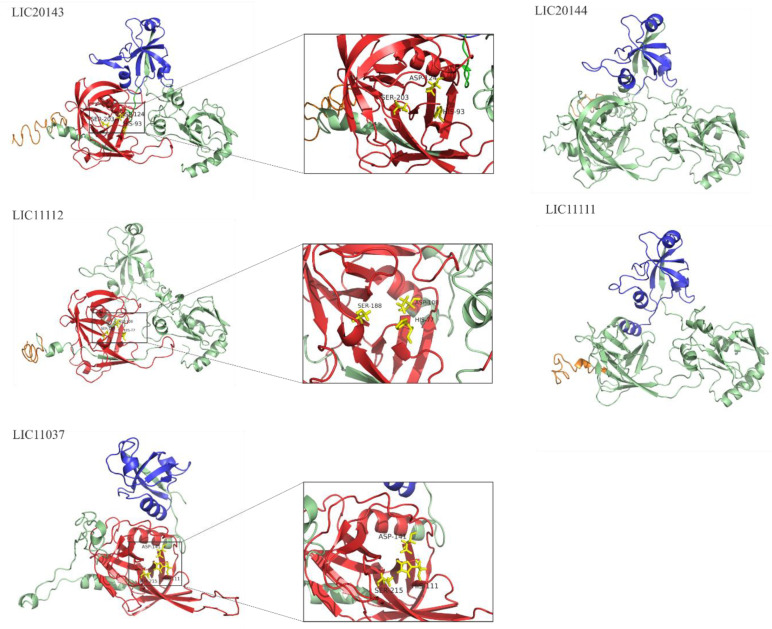
3D theorical model of LIC20143, LIC20144, LIC11112, LIC11111, and LIC11037 from *L. interrogans* serovar Copenhageni. The 3D model was generated by I-TASSER and visualized by PyMOL. The regions in red represent the serine protease domain, in blue the PDZ domain and in orange the signal peptide region. The catalytic triad (histidine, aspartate, and serine; HDS) is represented as yellow sticks with the correspondent position number of the residue. The regions in green do not have any known function.

**Figure 5:**
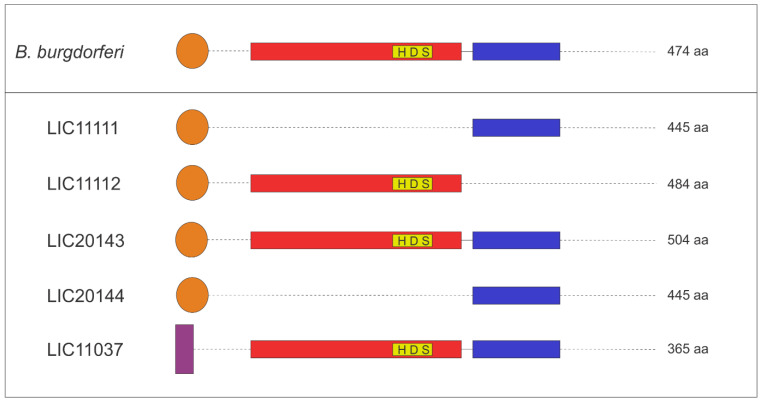
Domain sequence architecture. Schematic representation of the domain organization of the five proteins discussed here. HtrA from *B. burgdorferi* used as a reference showing the signal peptide, serine protease and PDZ domains, common to this family of proteins. The regions in red represent the serine protease domain, in blue the PDZ domain, in orange the signal peptide region and in purple the transmembrane region. The catalytic triad (histidine, aspartate, and serine; HDS) is in the yellow box.

3D structure prediction of LIC11037, LIC20143, LIC20144, LIC11111, and LIC11112 proteins was modeled using the I-TASSER web server. The Figure 4 was generated using the PyMOL program. The proteolytic domains are shown in red, while PDZ domains are in blue. The signal peptide is shown in orange, and the regions with unknown functions are in green. The active triad is indicated by yellow sticks. The serine protease domain is present in LIC20143, LIC11112, and LIC11037 and is zoomed in for better visualization of the three amino acid residues responsible for the active region: a histidine (His), an aspartic acid (Asp) and a serine (Ser) residue, as described previously [20]. LIC20143 has His-93, Asp-124, and Ser-203; LIC11112 His-77, Asp-108 and Ser-188; and LIC11037 His-111, Asp-141, and Ser-215. Only the LIC11112 protein lacks the PDZ domain according to the in silico analyses. The domain architecture for all proteins is illustrated in Figure 5 and is based on the organization of HtrABb, used as a reference of HtrA. The color of the scheme follows the same pattern shown in the 3D structure model (Figure 4), except for LIC11037, which has a transmembrane region depicted in purple. 

### 3.5. Operon Properties

**Figure 6:**
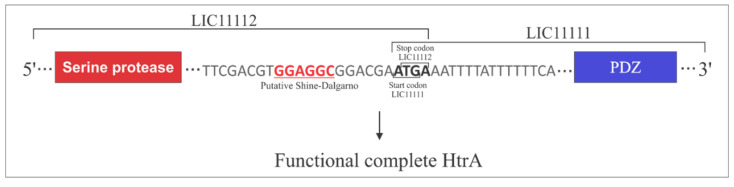
Operon properties. LIC11112 and LIC11111 are presented in an operon. The regions of the serine protease and PDZ domains are indicated in the red and blue box, respectively. The stop codon of LIC11112 and start codon of LIC11111 are indicated, with the putative Shine–Dalgarno sequence highlighted.

LIC11111 and LIC11112 occur in an operon together with two more genes, LIC11110 and LIC11113, these two genes are annotated as histidine kinase response regulator hybrid protein and hypothetical protein, respectively. LIC20143 and LIC20144 are also in operon together with LIC20142, annotated as adenine phosphoribosyl transferase. Since LIC11111 only has the PDZ domain and LIC11112 only has serine protease, the fact that they occur in an operon together suggests the organization of a complete functional HtrA heterodimer. This proposal is illustrated in Figure 6, in which the stop codon (TGA) at the C-terminal of LIC11112 ends with the beginning of the start codon (ATG) at the N-terminal of LIC11111. Serine protease and PDZ domains are depicted in LIC11112 and LIC11111, respectively. Also shown is a putative Shine–Dalgarno sequence upstream LIC11112. “Termination reinitiating” (TeRe) was reported to mainly occur in eukaryotes [43] but also in bacteria [44].

### 3.6. Search for Serine Protease Domain by Alignment

Besides whole-protein alignment, the sequence of serine protease domain alone was investigated among the 20 species of *Leptospira* selected. The search for similarities of the catalytic triad was performed because in silico analysis showed that three of the five proteins studied here possessed a serine protease domain. To better investigate the amino acids responsible for the catalytic triad, an alignment was performed. Figures S4–S6 show the alignment highlighting the three amino acids responsible for the serine protease domain: His-Asp-Ser (H-D-S) residues for the LIC20143, LIC11112, and LIC11037 proteins, respectively. These amino acids are conserved in all 20 selected species, from pathogenic to saprophytic, for the three proteins.

### 3.7. Serine Protease Domain Alignment for LIC20143, LIC11112, and LIC11037

**Figure 7:**
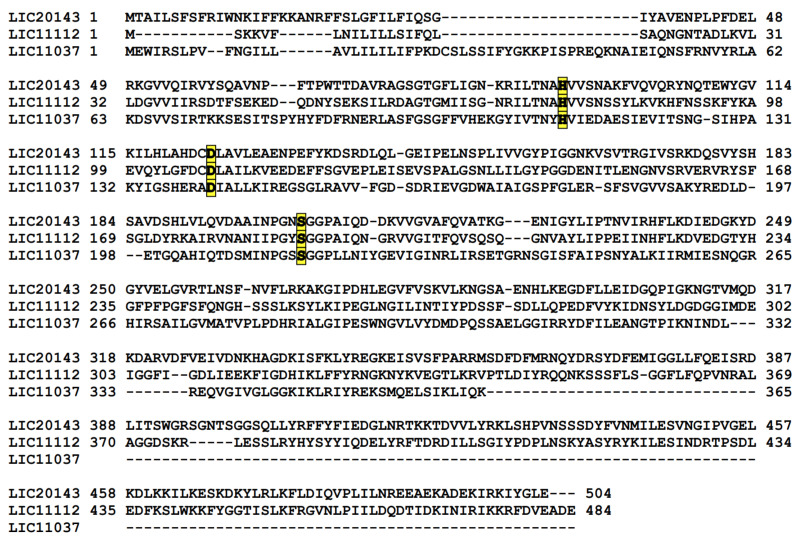
Alignment of the LIC20143, LIC11112, and LIC11037 of *L. interrogans* serovar Copenhageni. Alignment was done using the PROMALS3D tool, which aligns the sequences considering the amino acid sequence and protein domains. The amino acids histidine (H), aspartic acid (D) and serine (S) are highlighted showing that they are conserved in these three proteins.

After we searched for conserved amino acids belonging to the serine protease domain and discovered that LIC20143, LIC11112, and LIC11037 have the catalytic triad, we did an alignment to see if these three proteins could be aligned one with the other. We found that the triad is conserved between these sequences, even though the rest of the coding sequence is diverse (Figure 7).

## 4. Discussion 

The role of HtrA in bacterial virulence has been linked to increased fitness of pathogens due to resistance against stress conditions during infection. In recent years, it was discovered that HtrAs could also be involved in multiple host-pathogen interactions because these proteins can be secreted by the bacteria into the environment [25,45,46]. These extracellular proteins can target surface proteins of host cells to promote virulence by processing adhesins and ECM components [47]. Proteases of the HtrA family are found in multiple microbes, including archaea, Gram-negative and Gram-positive bacteria [48]. HtrA is essential for the virulence of various pathogens including *Yersinia enterocolitica*, *Klebsiella pneumoniae, Chlamydia trachomatis*, *Salmonella enterica*, *Listeria monocytogenes*, *Legionella pneumophila*, *Shigella flexneri, Burkholderia cenocepacia*, and *B. burgdorferi* [49,50,51,52,53,54,55,56]. It has been reported that the inactivation of HtrA proteins in *S. typhimurium*, *K. pneumoniae*, and *Streptococcus pneumoniae* reduces bacterial virulence in experimental animals [57,58]. Interestingly, HtrA protein from *S. flexneri* (DegP) has demonstrated an important role in acid resistance, since it needs to survive in human stomach and, therefore, has been fundamental for an efficient intercellular spread [54]. On the other hand, HtrA from *L. monocytogenes* has been shown as an important factor in biofilm formation [56]. 

*E. coli* have three genes annotated as proteases of the HtrA family (degP, degQ, degS) [59,60], while *L. interrogans* serovar Copenhageni have five genes related to the HtrA family. The genome of *Leptospira* spp. consists of two circular chromosomes and they have higher replicon content variation when compared to other spirochaetes. Possibly, this variability is associated with their ability to adapt in a wide range of hosts and environments [61]. As example, *L. borgpetersenii* has a smaller genome compared to *L. interrogans* and, although they causes similar clinical symptoms in infections, the transmission modes are different [62]. *L. borgpetersenii* does not need some genes that are important to live in an external environment, because the transmission of *L. borgpetersenii* occurs via direct host-to-host contact. Interestingly, the five genes studied here share high similarity with *L. borgpetersenii,* suggesting that HtrA could be more active in the host infections.

Genome data mining constitutes a rich source of information regarding putative virulence factors, including proteases. The annotated HtrA sequences in the *L. interrogans* serovar Copenhageni genome: LIC20143 and LIC20144, both found in an operon in chromosome II, LIC11111 and LIC11112, likewise in an operon, but in chromosome I, and LIC11037. As LIC11111 codes for a protein containing only a PDZ domain, we hypothesize that the co-expression of the serine protease coded by LIC11112 could render a functional heterodimer, constituting a complete HtrA holoenzyme (as shown in Figure 6). We suggest that a different translation coupling mechanism, namely TeRe, could be the way in which these two genes are translated [63]. This mechanism has been mainly studied in eukaryotic RNA viruses, where the same ribosome (or at least the same small subunit) that terminates an upstream gene initiates translation of a nearby or overlapping downstream gene [43,64]. In bacteria, it is not known whether the small subunit or the large subunit of the ribosome is involved, but TeRe has been proposed to operate [44]. LIC11112 and LIC11111 have a four-nucleotide overlap (ATGA), consistent with the TeRe mechanism, especially because the downstream of LIC11112, there is a putative Shine–Dalgarno (SD) sequence, which has been proposed to play an important role in the reinitiating step. Although LIC20143 has both PDZ and serine protease domains, as LIC20144 has only PDZ, perhaps the co-expression of LIC20143 coded serine protease may be involved in some regulatory mechanism that would help to form a functional molecule, but this hypothesis remains to be investigated.

Comparable sequences to HtrA proteins are found in all 20 leptospiral species studied here, and it is worth mentioning that despite their genome annotation, only LIC20143 and LIC11037 possess both PDZ and serine protease domains, typical of HtrA proteins. PDZ is a domain responsible for protein–protein interaction, and it is usually about 90 residues with 5-6 β-strands and 2 α-helixes [65,66,67,68,69]. The three proteins that we found to possess this domain have the same characteristics in terms of size and secondary structures.

According to in silico analyses, the proteins are highly conserved among pathogenic strains. Since HtrAs are potentially important in bacterial dissemination and actively contribute to the release of inflammatory cytokines in *B. burgdorferi* infection [47], it is possible that HtrA proteins of *Leptospira* could contribute to bacterial virulence. Primarily, HtrA proteins display chaperone and processing roles [70]. For instance, borrelial HtrA (HtrAB, BB0104) is an immunogenic protease whose fundamental structural unit is a trimer and has various proteolytic and chaperone functions [71]; HtrABb selectively degrades several endogenous proteins, including the virulence factor BB0323, which is processed by HtrABb into two N- and C-terminal peptides [72]. It has been demonstrated that the presence of functional HtrA protein is necessary for various bacterial species to withstand stressful conditions, such as heat shock or oxidative stress [59,73,74]. It has been shown that HtrA protein is important for survival in the host and/or virulence of several bacterial pathogens such as *S. enterica* serovar Typhimurium [53], *L. monocytogenes* [56], *K. pneumoniae* [50], and *Y. enterocolitica* [52]. 

High-throughput transcriptome data based on next-generation sequencing (NGS) has revealed how leptospiral CDSs respond when encountering environmental/host cues. RNAseq data obtained from virulent *L. interrogans* intraperitoneally implanted in rats by dialysis membrane chamber (DMC), in comparison to the in vitro cultivated virulent strain, revealed important transcriptome changes in the microenvironment that most approaches the in vivo infection [74]. LIC11111 and LIC11112 were upregulated comparably in DMC (1.81- and 1.84-fold change, respectively), once again suggesting co-transcription; furthermore, both proteins were detected in a quantitative proteome study [75]. High levels of LIC11037, LIC20143, and LIC20144 transcripts were found in DMC-containing virulent leptospires [74], and like LIC11111 and LIC11112, were also detected and quantified in proteomics study [75], suggesting that these HtrA proteins could participate in infection. 

Despite their putative intracellular chaperone roles, it has become evident that HtrA proteins are involved in the pathogenesis of various microorganisms, targeting and degrading host molecules, independently of their cellular location. The serine protease domains that we found in silico contain the conserved catalytic triad, suggesting that the leptospiral HtrA could be involved in degrading host components during infection, contributing to bacterial virulence. In *H. pylori*, a functional HtrA is able to cleave components of the epithelial intercellular junction, such as E-cadherin [26] and claudin-8 [76], favoring the crossing of the epithelial barrier upon infection. Several bacteria have been reported to transport HtrA to the extracellular space, including Gram-negative and Gram-positive pathogens such as *H. pylori*, *Campylobacter jejuni*, *B. burgdorferi*, *Bacillus anthracis*, and *Chlamydia* species [25,45,77]. However, how these proteins cross the bacterial outer membrane has not yet been elucidated [48]. Eshghi and colleagues [78] explored the composition and quantity of exoproteins of pathogenic *L. interrogans*. The authors were able to identify both LIC20143 and LIC20144 proteins as being secreted. LIC11111, LIC11112, and LIC11037 proteins could not be identified, which could have been due to the lower protein abundance in relation to the latter two [75]. Thus, the exact cellular location of these proteins remains to be determined experimentally. 

Rapid penetration, tissue invasion, and dissemination of virulent leptospires in susceptible hosts are major determinants for the success of infection. Mechanisms to overcome host barriers, such as ECM and immune defense, including the complement system, can ensure the prompt establishment of leptospires in target organs, predominantly kidneys [3]. The kinetics of leptospiral infection in hamsters revealed that bacteria could be detected in all organs 1 hour after intraperitoneal inoculation [4]. These results suggest a potent proteolytic activity associated with this pathogen. However, the way in which HtrA proteins are involved in this activity remains to be elucidated by in vitro and in vivo studies. Taken together, our in silico analysis points out that these five proteins, comparable to their counterparts in other pathogens, may have a role in leptospiral pathogenesis, most probably by degrading host components. These findings warrant further bench studies to better characterize these HtrA proteins. 

## Figures and Tables

**Table 1:** Features of HtrAs Coding Sequences.

Gene Locus	Strain	Cleavage Sites	Sequence	Transmembrane Region	Serine Protease	PDZ Domains	Size (aa)	Cellular Localization
LIC11111	*L. interrogans*	24–25	VPLKA/ETILV	-	-	175–280	445	Outer membrane
	*L. borgpetersenii*	31–32	GPLKA/ETILV	-	-	182–287	452	Outer membrane
	*L. licerasiae*	23–24	ENVEA/KADSE	-	-	206–293	458	Outer membrane
	*L. biflexa*	22–23	SNLIA/EEFED	-	-	-	454	Outer membrane
LIC11112	*L. interrogans*	20–21	FQLSA/QNGNT	-	55–230	-	484	Outer membrane
	*L. borgpetersenii*	23–24	FHLPA/QNGNS	Inside (1–4)	32–230	-	482	Outer membrane
				TMhelix (5–24)				
				Outside (25–482)				
	*L. licerasiae*	21–22	FPVFS/QTNGN	-	-		489	Outer membrane
	*L. biflexa*	20–21	HSAFS/QTESE	-	-	237–338	485	Outer membrane
LIC11037	*L. interrogans*	-		Inside (1–6) TMhelix (7–24) Outside (25–365)	68–256	270-353	365	Outer membrane
	*L. borgpetersenii*	-		Inside (1–6) TMhelix (7–26) Outside (27–368)	71–259	273-356	368	Outer membrane
	*L. licerasiae*	-		Inside (1–8) TMhelix (9–31) Outside (32–366)	69–257	271-354	366	Outer membrane
	*L. biflexa*	33–34	EIRSA/VTKLF	Inside (1–6) TMhelix (7–29) Outside (30–375)	72-260	289–357	375	Inner membrane
LIC20143	*L. interrogans*	38–39	SGIYA/VENPL	Inside (1–19) TMhelix (20–39) Outside (40–504)	56–243	252–345	504	Outer membrane
	*L. borgpetersenii*	-			54–241	258-343	502	Outer membrane
	*L. licerasiae*	21–22	LPLFS/EERSD		29-225	232–327	486	Outer membrane
	*L. biflexa*	51–52	STSHA/EPNGQ	Inside (1–26) TMhelix (27–49) Outside (50–515)	69-249	265–356	515	Outer membrane
LIC20144	*L. interrogans*	19–20	FEVLA/EISSK	-	-	263–343	527	Outer membrane
	*L. borgpetersenii*	19–20	LGVFA/EKVES	-	-	263–343	525	Outer membrane
	*L. liceraseae*	21–22	SGVSA/KKPKP	-	-	243–323	508	Outer membrane
	*L. biflexa*	-	-	-	-	-	490	Cytoplasmatic

## Supplementary Materials

The following are available online at https://www.mdpi.com/2414-6366/5/4/179/s1, Figure S1: Amino acid sequence identities of LIC11111 from 20 different *Leptospira* strains. The image was generated comparing the sequence of the protein encoded by LIC11112 from *L. interrogans* serovar Copenhageni with other 20 species (pathogenic, intermediate and saprophytic). Similarity is shown in red above 80%, in yellow between 50% and 80% and in green below 50%. Figure S2: Amino acid sequence identities of LIC11037 from 20 different *Leptospira* strains. The image was generated comparing the sequence of the protein encoded by LIC11112 from *L. interrogans* serovar Copenhageni with other 20 species (pathogenic, intermediate and saprophytic). Similarity is shown in red above 80%, in yellow between 50% and 80% and in green below 50%.; Figure S3: Amino acid sequence identities of LIC20144 from 20 different *Leptospira* strains. The image was generated comparing the sequence of the protein encoded by LIC11112 from *L. interrogans* serovar Copenhageni with other 20 species (pathogenic, intermediate and saprophytic). Similarity is shown in red above 80%, in yellow between 50% and 80% and in green below 50%. Figure S4: Alignment of the LIC20143 of *L. interrogans* serovar Copenhageni with other *Leptospira* species. Alignment was done using the PROMALS3D tool, which aligns the sequences considering the amino acid sequence and protein domains. The amino acids Histidine (H), Aspartic acid (D) and Serine (S)–highlighted-are present in all 20 species of *Leptospira* selected in this study and represent the residues essential to the catalytic activity. Figure S5: Alignment of the LIC11112 of *L. interrogans* serovar Copenhageni with other Leptospira species. Alignment was done using the PROMALS3D tool, which aligns the sequences considering the amino acid sequence and protein domains. The amino acids Histidine (H), Aspartic acid (D) and Serine (S)–highlighted-are present in all 20 species of *Leptospira* selected in this study and represent the residues essential to the catalytic activity. Figure S6: Alignment of the LIC11037 of *L. interrogans* serovar Copenhageni with other *Leptospira* species. Alignment was done using the PROMALS3D tool, which aligns the sequences considering the amino acid sequence and protein domains. The amino acids Histidine (H), Aspartic acid (D) and Serine (S)–highlighted-are present in all 20 species of *Leptospira*, except the Histidine that it is not present in *L. vanthielle*, selected in this study and represent the residues essential to the catalytic activity. Table S1: NCBI Accession IDs of all CDS for LIC11111, LIC11112, LIC20143, LIC20144 and LIC11037. They are displayed as follows: in red the pathogenic strains, in blue the intermediate ones and in green the saprophyte. All CDS were retrieved from the NCBI database through the Basic Local Alignment Search Tool (BLASTp).
