# Supplementary material for: In Silico Structural and Functional Characterization of HtrA Proteins of Leptospira spp.: Possible Implications in Pathogenesis
Source: Trop Med Infect Dis. 2020 Nov 28;5(4):179. doi: 10.3390/tropicalmed5040179 (PMC7709667; doi:10.3390/tropicalmed5040179)
Supplement: Supplementary file 1 [file tropicalmed-05-00179-s001.pdf]

Tropical Medicine and Infectious Disease

	L. interrogans	L. noguchii	L. kirschneri	L. alstonii	L. weilii	L. alexanderi	L. borgpetersenii	L. santarosai	L. kmetyi	L. fainei	L. broomii	L. wolffii	L. licerasiae	L. inadai	L. wolbachii	L. yanagawae	L. vanthielii	L. terpstrae	L. meyeri	L. biflexa
L. interrogans																				
L. noguchii	96																			
L. kirschneri	97	96																		
L. alstonii	81	80	81																	
L. weilii	82	81	82	82																
L. alexanderi	81	80	81	83	97															
L. borgpetersenii	81	80	82	82	92	94														
L. santarosai	81	81	81	82	90	90	89													
L. kmetyi	78	76	78	78	79	79	78	76												
L. fainei	48	47	48	48	48	48	46	47	48											
L. broomii	47	46	47	46	47	47	45	45	46	92										
L. wolffii	49	49	48	48	47	47	45	47	47	68	69									
L. licerasiae	46	47	46	46	48	48	47	46	47	69	69	80								
L. inadai	46	47	46	46	47	47	45	46	46	92	94	69	70							
L. wolbachii	34	34	34	35	34	34	34	35	35	37	36	36	35	37						
L. yanagawae	35	35	35	33	35	35	35	36	33	35	35	35	34	37	80					
L. vanthielii	33	33	34	34	34	33	33	35	35	37	36	36	33	36	98	79				
L. terpstrae	33	33	33	33	34	34	34	35	34	36	36	36	34	37	93	80	93			
L. meyeri	34	34	34	35	35	35	34	34	33	37	36	35	33	36	92	77	93	90		
L. biflexa	36	36	36	33	35	35	35	35	35	33	35	37	34	35	81	87	79	80	78	

Figure S1. Amino acid sequence identities of LIC11111 from 20 different *Leptospira* strains. The image was generated comparing the sequence of the protein encoded by LIC11112 from *L. interrogans* serovar Copenhageni with other 20 species (pathogenic, intermediate and saprophytic). Similarity is shown in red above 80%, in yellow between 50% and 80% and in green below 50%.

	L. interrogans	L. noguchii	L. kirschneri	L. alstonii	L. weilii	L. alexanderi	L. borgpetersenii	L. santarosai	L. kmetyi	L. fainei	L. broomii	L. wolffii	L. licerasiae	L. inadai	L. wolbachii	L. yanagawae	L. vanthielii	L. terpstrae	L. meyeri	L. biflexa
L. interrogans																				
L. noguchii	98																			
L. kirschneri	99	98																		
L. alstoni	91	93	92																	
L. weilii	92	93	92	96																
L. alexanderi	88	89	88	91	90															
L. borgpetersenii	88	89	89	90	89	95														
L. santarosai	88	89	89	91	89	93	93													
L. kmetyi	91	92	91	94	92	92	92	92												
L. fainei	78	79	78	78	78	77	78	78	80											
L. broomii	78	79	78	78	78	76	78	77	80	99										
L. wolffii	77	78	77	77	78	75	76	75	78	87	87									
L. licerasiae	76	78	77	75	78	73	75	73	76	84	84	90								
L. inadai	77	79	78	78	77	76	77	77	79	99	99	86	84							
L. wolbachii	61	60	61	61	61	60	60	60	61	60	60	60	62	60						
L. yanagawae	61	60	60	61	61	60	60	60	61	60	60	59	61	60	95					
L. vanthielii	61	60	61	61	61	60	60	60	61	60	60	60	62	60	100	95				
L. terpstrae	61	60	61	61	61	60	60	60	61	60	60	60	62	60	99	95	99			
L. meyeri	60	60	60	61	60	60	60	60	60	59	59	59	61	59	99	95	99	99		
L. biflexa	60	60	60	60	61	60	60	60	60	59	59	59	61	59	96	97	96	96	96	

Figure S2. Amino acid sequence identities of LIC11037 from 20 different *Leptospira* strains. The image was generated comparing the sequence of the protein encoded by LIC11112 from *L. interrogans* serovar Copenhageni with other 20 species (pathogenic, intermediate and saprophytic). Similarity is shown in red above 80%, in yellow between 50% and 80% and in green below 50%.

	L. interrogans	*L. noguchii*	*L. kirschneri*	*L. alstonii*	*L. weilii*	*L. alexanderi*	*L. borgpetersenii*	*L. santarosai*	*L. kmetyi*	*L. fainei*	*L. broomii*	*L. wolffii*	*L. licerasiae*	*L. inadai*	*L. wolbachii*	*L. yanagawae*	*L. vanthielii*	*L. terpstrae*	*L. meyeri*	*L. biflexa*
L. interrogans																				
L. noguchii	91																			
L. kirschneri	93	93																		
L. alstonii	83	84	84																	
L. weilii	83	83	85	92																
L. alexanderi	83	84	84	89	96															
L. borgpetersenii	84	84	84	89	92	94														
L. santarosai	79	83	85	87	86	86	85													
L. kmetyi	81	81	82	82	82	82	82	77												
L. fainei	57	56	58	56	55	55	58	57	59											
L. broomii	54	56	53	53	54	54	54	56	57	93										
L. wolffii	55	56	56	55	57	55	57	57	58	69	70									
L. licerasiae	54	55	56	56	53	54	56	55	54	65	64	73								
L. inadai	59	56	54	54	54	53	53	56	55	92	95	72	63							
L. wolbachii	37	37	37	37	37	37	36	37	37	37	38	39	38	38						
L. yanagawae	37	36	36	35	37	37	37	37	36	38	38	38	37	37	76					
L. vanthielii	37	36	36	37	36	36	37	37	36	38	38	38	38	38	95	77				
L. terpstrae	38	37	37	37	37	37	37	38	36	37	39	39	38	39	91	76	90			
L. meyeri	38	37	37	37	37	37	37	37	37	38	39	39	37	39	86	75	86	88		
L. biflexa	37	36	36	35	36	36	35	36	36	37	38	38	38	38	74	80	75	74	73	

Figure S3. Amino acid sequence identities of LIC20144 from 20 different *Leptospira* strains. The image was generated comparing the sequence of the protein encoded by LIC11112 from *L. interrogans* serovar Copenhageni with other 20 species (pathogenic, intermediate and saprophytic). Similarity is shown in red above 80%, in yellow between 50% and 80% and in green below 50%.

L. interrogans	LIGNKRILTNA**H**VVSN	AHDC**D**LAVLEAEN	NPGN**S**GGPAIQD
L. noguchii	LIGNKRILTNA**H**VVSN	AHDC**D**LAVLEAEN	NPGN**S**GGPAIQD
L. kirshneri	LIGNKRILTNA**H**VVSN	AHDC**D**LAVLEAEN	NPGN**S**GGPAIQD
L. alstonii	LIGNKRILTNA**H**VVSN	AHDC**D**LAVLEAES	NPGN**S**GGPAIQD
L. weilii	LIGNKRILTNA**H**VVSN	AHDC**D**LAVLEAES	NPGN**S**GGPAIQD
L. alexanderi	LIGNKRILTNA**H**VVSN	AHDC**D**LAVLEAES	NPGN**S**GGPAIQD
L. borgpetersenii	LIGNKKILTNA**H**VVSN	AHDC**D**LAVLEAES	NPGN**S**GGPAIQD
L. santarosai	LIGNKRILTNA**H**VVSN	AHDC**D**LAVLEAES	NPGN**S**GGPAIQD
L. kmetyi	LIGNKRILTNA**H**VVSN	AHDC**D**LAVLEAES	NPGN**S**GGPAIQD
L. fainei	LIGKKRILTNA**H**VVSN	AHDC**D**LAILEAED	NPGN**S**GGPAIQG
L. broomii	LIGKKRILTNA**H**VVSN	AHDC**D**LAILEAED	NPGN**S**GGPAIQD
L. wolffi i	LIGNKRILTNA**H**VISN	AHDC**D**LAVLEAED	NPGN**S**GGPAIQN
L. licerasiea	LIGNKRILTNA**H**VISN	AHDC**D**LAVLEAED	NPGN**S**GGPAIQN
L. inadai	LIGKKRILTNA**H**VVSN	AHDC**D**LALLEAED	NPGN**S**GGPAIQD
L. wolbachi	LISKNRILTNA**H**VVSN	AHDC**D**LALLEVSD	NPGN**S**GGPALQN
L. yanagawe	IISKNRILTNA**H**VVSN	AHDC**D**LAILEVPD	NPGN**S**GGPALQD
L. vanthielli	LISKNRILTNA**H**VVSN	AHDC**D**LALLEVSD	NPGN**S**GGPALQN
L. terpstrae	LISKNRILTNA**H**VVSN	AHDC**D**LALLEVSD	NPGN**S**GGPALQN
L. meyeri	LISKNRILTNA**H**VVSN	AHDC**D**LALLEVSD	NPGN**S**GGPALQE
L. biflexa	IIAKNRILTNA**H**VVSN	AHDC**D**LAILEVPD	NPGN**S**GGPALQN

Figure S4. Alignment of the LIC20143 of *L. interrogans* serovar Copenhageni with other *Leptospira* species. Alignment was done using the PROMALS3D tool, which aligns the sequences considering the amino acid sequence and protein domains. The amino acids Histidine (H), Aspartic acid (D) and Serine (S)—highlighted—are present in all 20 species of *Leptospira* selected in this study and represent the residues essential to the catalytic activity.

L. interrogans	ISGNRILTNA**H**VVSNSSY	YLGFDC**D**LAILKVEE	VNANIIPGY**S**GGPAIQNGR
L. noguchii	ISGNRILTNA**H**VVSNSSY	YLGFDC**D**LAILKVEE	VNANIIPGY**S**GGPAIQNGK
L. kirshneri	ISGNRILTNA**H**VVSNSSY	YLGFDC**D**LAILKVEE	VNANIIPGY**S**GGPAIQNGR
L. alstonii	ISGNRILTNA**H**VVSNSGY	YLGFDC**D**LAILKVEE	VNANIIPGY**S**GGPAIQNGK
L. weillii	ISGNRILTNA**H**VVSNSGY	YLGFDC**D**LAILKVEE	VNANIIPGY**S**GGPAIQNGK
L. alexanderi	ISGNRILTNA**H**VISNSSY	FLGFDC**D**LAILKVEE	VNANIIPGY**S**GGPAIQNGK
L. borgpetersenii	ISGNRILTNA**H**VVSNSNY	YLGFDC**D**LAILKVEE	VNANIIPGY**S**GGPAIQSGK
L. santarosai	ISGNRILTNA**H**VVSDSSY	YLGFDC**D**LAILKVEE	VNANIIPGY**S**GGPAIQNGK
L. kmetyi	IAGNRILTNA**H**VVSNSGY	YLGFDC**D**LAILKVEE	VNANIVPGY**S**GGPAIQNGK
L. fainei	IQGNRILTNA**H**VVAESKF	FLGFDC**D**LALLKVED	VTANILPGY**S**GGPAIQNGK
L. broomii	IQGNRILTNA**H**VVAESKF	FLGFDC**D**LALLKVED	VTANILPGY**S**GGPAIQNGQ
L. wolffii	VAGNRILTNA**H**VISESKY	FLGFDC**D**LALIKVED	VGANILPGY**S**GGPAIQNGK
L. licerasiae	IAGNRILTNA**H**VISESKY	FIGFDC**D**LALISVED	VGANILPGY**S**GGPAIQNGK
L. inadai	IQGNRILTNA**H**VVAESKF	FLGFDC**D**LALLKVAD	VTANILPGY**S**GGPAVQNGQ
L. wolbachii	LPNQTILTNA**H**VVRDAKR	FIGYDC**D**LALLQVAD	ITANIQPGN**S**GGPAVQNGK
L. yanagawae	LPNQTILTNA**H**VVRDAKR	YIGYDC**D**LALLQVND	ISANIQPGN**S**GGPAVQNGK
L. vanthielli	LPNQTILTNA**H**VVRDAKR	FIGYDC**D**LALLQVTD	ITANIQPGN**S**GGPAVQNGK
L. terpstrae	LSNQTILTNA**H**VVRDAKR	FIGYDC**D**LALLQVTD	ITANIQPGN**S**GGPAVQNGK
L. meyeri	LPNQTILTNA**H**VVRDAKR	YIGYDC**D**LALLQVND	INANIQPGN**S**GGPAVQNGK
L. biflexa	LPNQTILTNA**H**VVRDAKR	FIGYDC**D**LALLQVND	ISANIQPGN**S**GGPAVQNGK

Figure S5. Alignment of the LIC11112 of *L. interrogans* serovar Copenhageni with other *Leptospira* species. Alignment was done using the PROMALS3D tool, which aligns the sequences considering the amino acid sequence and protein domains. The amino acids Histidine (H), Aspartic acid (D) and Serine (S)—highlighted—are present in all 20 species of *Leptospira* selected in this study and represent the residues essential to the catalytic activity.

L. interrogans	VHEKGYIVTNY**H**VIED	HERA**D**IALLKIRE	NPGS**S**GGPLLNI
L. noguchii	VHEKGYIVTNY**H**VIED	HERA**D**IALLKIRE	NPGS**S**GGPLLNI
L. kirshneri	VHEKGYIVTNY**H**VIED	HERA**D**IALLKIRE	NPGS**S**GGPLLNI
L. alstonii	VHEKGYIVTNY**H**VIEG	HERA**D**IALLKIRE	NPGS**S**GGPLLNI
L. weilii	VHEKGYIVTNY**H**VIEG	HERA**D**IALLKIRE	NPGS**S**GGPLLNI
L. alexanderi	VHEKGYIVTNY**H**VIEG	HERA**D**IALLKIRE	NPGS**S**GGPLLNI
L. borgpetersenii	VHEKGYIVTNY**H**VIEG	HERA**D**IALLKIRE	NPGS**S**GGPLLNI
L. santarosai	VHEKGYIVTNY**H**VIEG	HERA**D**IALLKIRE	NPGS**S**GGPLLNI
L. kmetyi	VHEKGYIVTNY**H**VIEG	HERA**D**IALLKIRE	NPGS**S**GGPLLNI
L. fainei	IHEKGYIVTNF**H**VIEG	HERA**D**ISLLKIRE	NPGS**S**GGPLLNI
L. broomii	IHEKGYIVTNF**H**VIEG	HERA**D**ISLLKIRE	NPGS**S**GGPLLNI
L. wolffii	IHEKGYIVTNF**H**VIQD	HERA**D**IALLKIKE	NPGS**S**GGPLLNI
L. licerasiea	IHEKGYVVTNF**H**VISD	HERA**D**IALLKIKE	NPGS**S**GGPLLNI
L. inadai	IHEKGYIATNF**H**VIEG	HERA**D**ISLLKIRE	NPGS**S**GGPLLNI
L. wolbachii	IDERGFVVTNY**H**VIKN	HERA**D**IALLKIPS	NPGS**S**GGPLLNI
L. yanagawe	IDERGFVVTNY**H**VIKN	HERA**D**IALLKIPS	NPGS**S**GGPLLNI
L. vanthielli	-	HERA**D**IALLKIPS	NPGS**S**GGPLLNI
L. terpstrae	IDERGFVVTNY**H**VIKN	HERA**D**IALLKIPS	NPGS**S**GGPLLNI
L. meyeri	IDERGFVVTNY**H**VIKN	HERA**D**IALLKIPS	NPGS**S**GGPLLNI
L. biflexa	IDERGFVVTNY**H**VIKN	HERA**D**IALLKIPS	NPGS**S**GGPLLNI

Figure S6. Alignment of the LIC11037 of *L. interrogans* serovar Copenhageni with other *Leptospira* species. Alignment was done using the PROMALS3D tool, which aligns the sequences considering the amino acid sequence and protein domains. The amino acids Histidine (H), Aspartic acid (D) and Serine (S)—highlighted—are present in all 20 species of *Leptospira*, except the Histidine that it is not present in *L. vanthielle,* selected in this study and represent the residues essential to the catalytic activity.

Table S1. NCBI Accession IDs of all CDS for LIC11111, LIC11112, LIC20143, LIC20144 and LIC11037. They are displayed as follows: in red the pathogenic strains, in blue the intermediate ones and in green the saprophyte. All CDS were retrieved from the NCBI database through the Basic Local Alignment Search Tool (BLASTp).

	**NCBI Accession I.D.**				
	**LIC11111**	**LIC11112**	**LIC20143**	LIC20144	LIC11037
L. interrogans	WP_000675215.1	WP_000039575.1	WP_000125234.1	WP_000944569.1	WP_001976240.1
L. kirschneri	WP_004765384.1	WP_004765506.1	WP_082293370.1	WP_004757632.1	EKO60201.1
L. noguchii	WP_017214502.1	WP_061247727.1	WP_004452497.1	WP_004449692.1	WP_002245775.1
L. alstonii	WP_020774559.1	WP_020774560.1	WP_061248069.1	WP_020772440.1	WP_020773847.1
L. weilii	WP_061222824.1	WP_003008403.1	WP_061222378.1	WP_061222350.1	WP_002996538.1
L. alexanderi	WP_078128092.1	WP_010578769.1	WP_078128700.1	WP_078128701.1	WP_078129293.1
L. borgpetersenii	EMO64919.1	EMO65007.1	WP_026054861.1	WP_002732170.1	EMO62864.1
L. santarosai	WP_004478972.1	WP_004491925.1	WP_076637811.1	WP_046943593.1	WP_016753804.1
L. kmetyi	WP_123179212.1	WP_100736899.1	WP_100756608.1	WP_123180574.1	WP_040913868.1
L. fainei	WP_016549290.1	WP_016549246.1	WP_016551179.1	WP_016551249.1	WP_016547636.1
L. broomii	WP_010570309.1	WP_010570308.1	WP_010568511.1	WP_010568510.1	WP_010570471.1
L. wolffii	WP_016544738.1	WP_01654468.1	WP_100758755.1	WP_135701288.1	WP_0165440.1
L. licerasiae	WP_008591953.1	WP_008592017.1	WP_008589276.1	WP_135667780.1	WP_008595422.1
L. inadai	WP_010411362.1	WP_010411360.1	WP_010409673.1	WP_010409676.1	WP_010411939.1
L. wolbachii	WP_015681858.1	WP_015680886.1	WP_015682936.1	WP_015682854.1	EOQ97072.1
L. yanagawe	WP_015677123.1	WP_039928164.1	WP_039929592.1	WP_015679341.1	WP_015678530.1
L. biflexa	WP_012390123.1	WP_012390124.1	WP_041770204.1	WP_012476598.1	WP_012387918.1
L. vanthielii	WP_002988486.1	WP_002988746.1	WP_002980682.1	WP_002980637.1	EMY68206.1
L. terpstrae	WP_039937265.1	WP_002972837.1	WP_002975534.1	WP_002975543.1	WP_002973171.1
L. meyeri	WP_004786397.1	WP_020776352.1	WP_020777264.1	WP_004788280.1	EKJ86609.1

© 2020 by the authors. Licensee MDPI, Basel, Switzerland. This article is an open access article distributed under the terms and conditions of the Creative Commons Attribution (CC BY) license (http://creativecommons.org/licenses/by/4.0/).
